# Repeatability of pupil diameter measurements using three different topography devices

**DOI:** 10.1371/journal.pone.0290417

**Published:** 2023-08-18

**Authors:** Amr Saad, Johannes Steinberg, Andreas Frings

**Affiliations:** 1 Department of Ophthalmology, University Hospital of Duesseldorf, Duesseldorf, Germany; 2 Department of Ophthalmology, University Medical Center, Hamburg, Germany; 3 Zentrum Sehstärke, Hamburg, Germany; 4 Augenheilkunde & Augenlaser Zentrum PD Dr. med. A. Frings, Nuremberg, Germany; Saarland University, GERMANY

## Abstract

**Purpose:**

To evaluate the intra- and inter-device repeatability of pupil diameter measurements using three different devices in patients prior to corneal refractive surgery.

**Methods:**

We examined preoperative measurements from a total of 204 eyes (102 patients) scheduled for corneal refractive surgery at two private centers between July and December 2021. Three consecutive scans were performed with three different devices (Sirius anterior segment analyzer, Pentacam HR, IOLMaster 500) in the same session by the same examiner under standardized conditions. To assess the intra- and inter-device repeatability, we calculated the Intraclass Correlation Coefficient (ICC) and demonstrated results using Bland-Altman plots.

**Results:**

The measurement accuracy (intra-device repeatability) of Sirius and IOLMaster was comparable (ICC = 0.64 and 0.61, respectively), with almost no statistically significant differences. Sirius showed the highest measurement accuracy among the three devices. Pentacam measurements resulted in lower precision, with an ICC of 0.09. The agreement between the pairs of devices (inter-device repeatability) was low (wide LoA ranges, Table 5).

**Conclusion:**

In this study, the intra-device repeatability of Sirius and IOLMaster was higher than that of the Pentacam, although it did not achieve an optimal level across all three devices. The three devices examined cannot be used interchangeably.

## Introduction

Precise measurements are essential for successful outcomes in refractive surgery. Pupil diameter (PD) is one of the most critical values, especially for patients undergoing corneal refractive surgery, when it comes to optimal optical zone calculation. A pupil diameter that is too large or an ablation zone that is too small will inevitably result in significantly higher order aberrations (HOA) as well as a loss of contrast sensitivity. To reduce the occurrence of postoperative HOA, including halos and glare, the optical zone must be large enough [1, 2]. Therefore, it is important to precisely determine the pupil diameter, especially in young patients with a large pupil diameter.

To date, various non-contact instruments have been made available for clinical use and preoperative examination. Since different devices use different techniques, it is important to ensure a high level of consistency between them to achieve high quality standard and satisfactory visual results. Among these devices, the Pentacam (Oculus Inc., Wetzlar, Germany), the IOLMaster 500 (Carl Zeiss Meditec AG, Germany) and the Sirius anterior segment analyzer (Schwind Eye-Tech-Solutions, Kleinostheim, Germany) are currently in use. The precision of the different measuring devices can be evaluated by analyzing the repeatability of measurement results in the same individuals. Knowledge of the measurement accuracy is highly important for clinical application, especially in borderline cases.

On the one hand, it is important to evaluate the (intra-device) repeatability of individual device measurements. On the other hand, the agreement (inter-device) reproducibility between the different devices is crucial in terms of the interchangeability of these instruments. In this context, repeatability describes the variability of results measured in a short time interval.

Previous studies have evaluated the accuracy of anterior segment parameters such as corneal thickness and curvature. However, none of them focused on PD measurements. In our study, the intra-repeatability was evaluated in terms of test-retest accuracy and the inter-device repeatability was evaluated between the three different devices. The purpose of this study was to provide refractive surgeons with a basis for the preoperative evaluation of potential candidates with regards to PD assessment.

## Materials and methods

This retrospective study included preoperative measurements prior to trans-photorefractive keratectomy (PRK) or Femto-Laser in Situ Keratomeleusis (LASIK) of 204 eyes (102 patients) from July to December 2021. This study was approved by the local research ethics committee of the University of Duesseldorf (Study-Nr.: 2018–292) and complies with the Declaration of Helsinki. All patients gave informed written consent for the use of their routinely collected data for research purposes and all data were pseudonymized for tracking individual cases where appropriate. Only the Principal Investigator had access to the pseudonymization key. We included only adult patients (> 18 years old) and none had any ocular disease, previous ocular surgery or trauma, or general disorders affecting the eye.

We investigated the reliability of pupil diameter measurements using the following devices: a rotating Scheimpflug camera (Pentacam HR, Oculus Inc., Wetzlar, Germany), a Scheimpflug camera with a Placido disc‑based corneal topographer (Sirius, Schwind Eye-Tech-Solutions Ltd., Germany) and a partial coherence interferometer (IOLMaster 500, Carl Zeiss Meditec Inc., Germany). The Pentacam HR uses a high-resolution 180° rotating Scheimpflug camera for a three-dimensional representation of the anterior segment parameters, including the PD. The IOLMaster 500 is an optical biometer based on partial coherence interferometry, which made the use of ultrasound biometry unnecessary [3]. The Sirius combines two systems, the Scheimpflug camera with Placido disk‑based corneal topography, to assess both the anterior and posterior corneal surface parameters.

Three consecutive measurements were performed with each of the three devices by an experienced examiner under the same standardized scotopic room conditions (approx. 1 Lux) for each device to minimize artificial variations in the results. Following a two-minute dark adaptation period, during which patients were instructed to keep both eyes open and avoid direct exposure to light, we began with the measurement of the right eye and subsequently switched to the left eye for each device. Patients had a short break between each measurement for tear film recovery. They were asked to blink between measurements and then fixate the target with their eyes wide open. Additionally, they were instructed gently to maintain a relaxed focus on the distance with their contralateral eye in the dark examination room. Only measurements with good quality (displayed by the respective device software) were used for analysis.

Statistical analysis was performed using R Core Team software (R Foundation for Statistical Computing, Vienna, Austria) from February to July 2022. Intra-device repeatability was assessed by calculating the Limits of Agreement (LoA) (defined as ±1.96SD), with a narrower LoA indicating higher agreement between measurements. Differences between individual scans and the mean of subject readings were visualized using an extended version of the Bland-Altman plot for multiple raters, as described by [4]. For each device, the Intraclass Correlation Coefficient (ICC) was calculated with two-way mixed effects, absolute agreement and single rater/measurement according to [5]. Differences between scan means were assessed using a mixed regression model in which each subject was considered a random effect and each scan a fixed effect. The differences between each pair of scans were assessed separately using the standard version of the Bland-Altman plot.

For the inter-device repeatability and interchangeability assessment of the three devices, we compared the first scan from each device and created a standard Bland-Altman plot for each pair of devices. We also calculated the ICC for each device pair using two-way mixed effects, absolute agreement and single rater/measurement. The ICC describes the consistency between repeated measurements and ranges from 0 to 1, with values above 0.9 indicating reasonable clinical accuracy.

## Results

To maintain a largely homogeneous and representative group composition, we randomly selected 102 patients for analysis from all preoperative measurements using a random number function of Excel (Microsoft Excel 2017, Microsoft^®^, Washington, USA).

**Fig 1:**
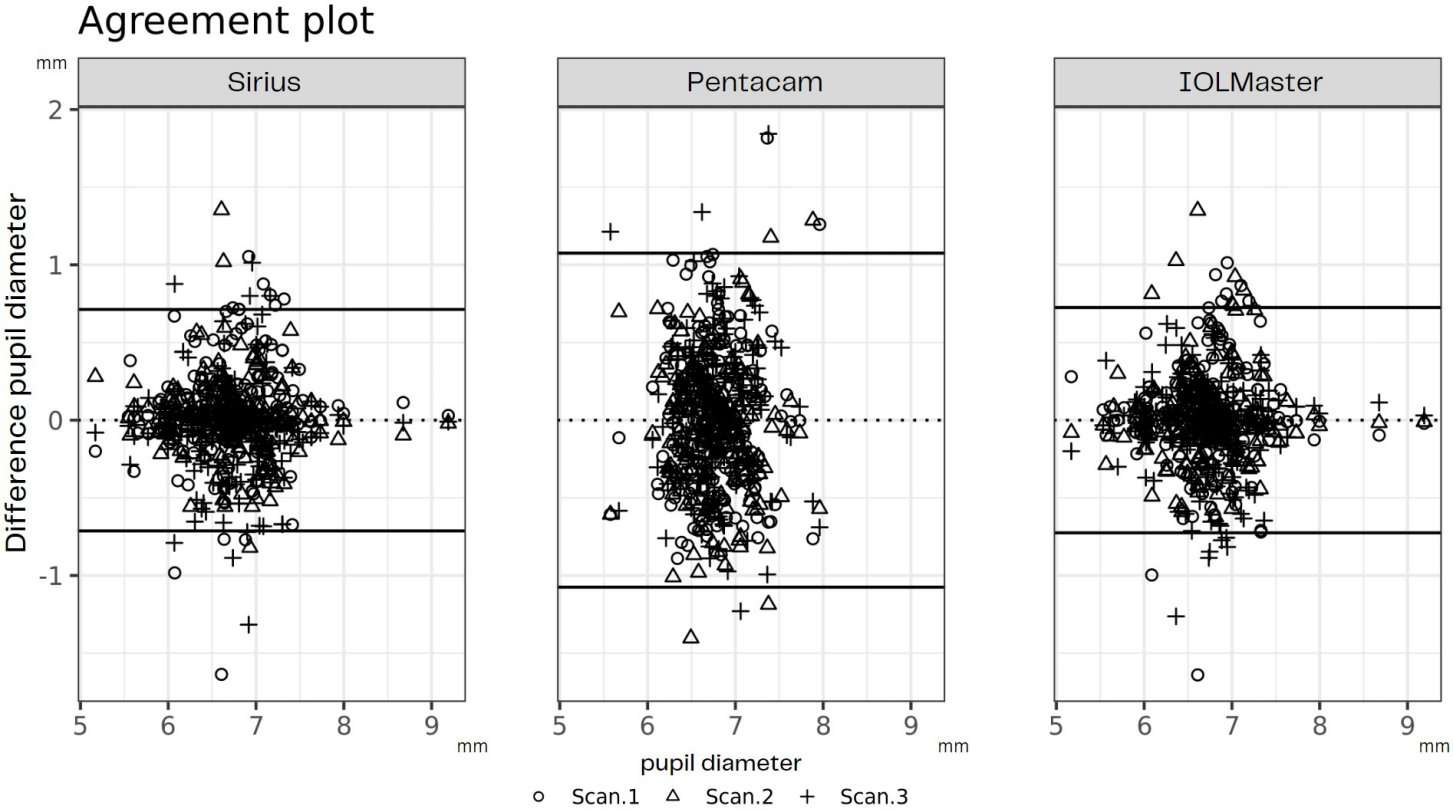
Agreement plot for intra-device repeatability of scan pairs of each device.

Fig 1 shows the intra-device repeatability of the three measurements of each device in the form of an agreement plot. Table 1 summarizes the within SD (Sw), ICC and LoA for each device, among others. Tables 2–4 show the repeatability values for the pair of scans for each device.

**Table 1:** Intra-device repeatability of each device.

Statistic	Sirius	Pentacam	IOLMaster
**N Eyes**	204	204	204
**N Measurements**	612	612	612
**Difference between scans, p-value**	0.048	0.264	0.126
**Overall mean (mm)**	6.728	6.771	6.731
**Between SD (mm)**	0.487	0.174	0.462
**Within SD (mm)**	0.364	0.549	0.370
**ICC [95% CI]**	0.643 [0.60;0.70]	0.093 [0.00;0,20]	0.61 [0.50;0.70]
**LoA (mm)**	-0.71,0.71	-1.08,1.08	-0.73,0.73

**SD**: standard deviation; **ICC**: Intraclass Coefficient; **CI**: Confidence Interval; **LoA**: Limits of Agreement

**Table 2:** Intra-device repeatability of IOLMaster scan pairs.

Statistic	Scan 1 vs Scan 2	Scan 1 vs Scan 3	Scan 3 vs Scan 3
**N Eyes**	204	204	204
**N Measurements**	408	408	408
**Mean difference**	0.036	0.075	0.039
**Difference between scans, p-value**	0.332	0.051	0.265
**Overall mean (mm)**	6.75	6.73	6.71
**Between SD (mm)**	0.47	0.45	0.47
**Within SD (mm)**	0.38	0.38	0.35
**ICC [95% CI]**	0.61 [0.51;0.69]	0.57 [0.47;0,66]	0.65 [0.56;0.72]
**LoA (mm)**	1.08,-1	1.14,-0.99	1.01,-0.93

**SD**: standard deviation; **ICC**: Intraclass Coefficient; **CI**: Confidence Interval; **LoA**: Limits of Agreement

**Table 3:** Intra-device repeatability of Sirius scan pairs.

Statistic	Scan 1 vs Scan 2	Scan 1 vs Scan 3	Scan 2 vs Scan 3
**N Eyes**	204	204	204
**N Measurements**	408	408	408
**Mean difference**	0.038	0.089	0.051
**Difference between scans, p-value**	0.268	0.032	0.117
**Overall mean (mm)**	6.75	6.73	6.71
**Between SD (mm)**	0.5	0.47	0.49
**Within SD (mm)**	0.34	0.41	0.33
**ICC [95% CI]**	0.68 [0.60;0.75]	0.56 [0.47;0,65]	0.69 [0.61;0.76]
**LoA (mm)**	0.99,-0.92	1.24,-1.06	0.96,-0.85

**SD**: standard deviation; **ICC**: Intraclass Coefficient; **CI**: Confidence Interval; **LoA**: Limits of Agreement

**Table 4:** Intra-device repeatability of Pentacam scan pairs.

Statistic	Scan 1 vs Scan 2	Scan 1 vs Scan 3	Scan 2 vs Scan 3
**N Eyes**	204	204	204
**N Measurements**	408	408	408
**Mean difference**	0.034	-0.054	-0.088
**Difference between scans, p-value**	0.515	0.352	0.095
**Overall mean (mm)**	6.75	6.79	6.77
**Between SD (mm)**	0.2	0.1	0.21
**Within SD (mm)**	0.53	0.58	0.53
**ICC [95% CI]**	0.12 [0.00;0.25]	0.03 [0.00;0,16]	0.13 [0.00;0.27]
**LoA (mm)**	1.5,-1.43	1.57,-1.67	1.38,-1.56

**SD**: standard deviation; **ICC**: Intraclass Coefficient; **CI**: Confidence Interval; **LoA**: Limits of Agreement

Regarding the intra-device repeatability of Sirius and IOLMaster, both showed a moderate ICC (ICC = 0.64 and 0.61, respectively), with Sirius showing a slightly higher accuracy. Sirius is the only device that showed a statistically significant difference between the scans (p < 0.05, Table 1). The confidence interval (CI) of both ICC values were similar, indicating that there was no statistically significant difference between the two instruments (Table 1). The Pentacam showed the lowest intra-device repeatability with an ICC of 0.09 and a CI outside that of Sirius and IOLMaster (Table 1). The small Sw of Sirius and IOLMaster (Sw = 0.36 and 0.37, respectively) and the high value of the Pentacam system (Sw = 0.55) also underline these results.

**Fig 2:**
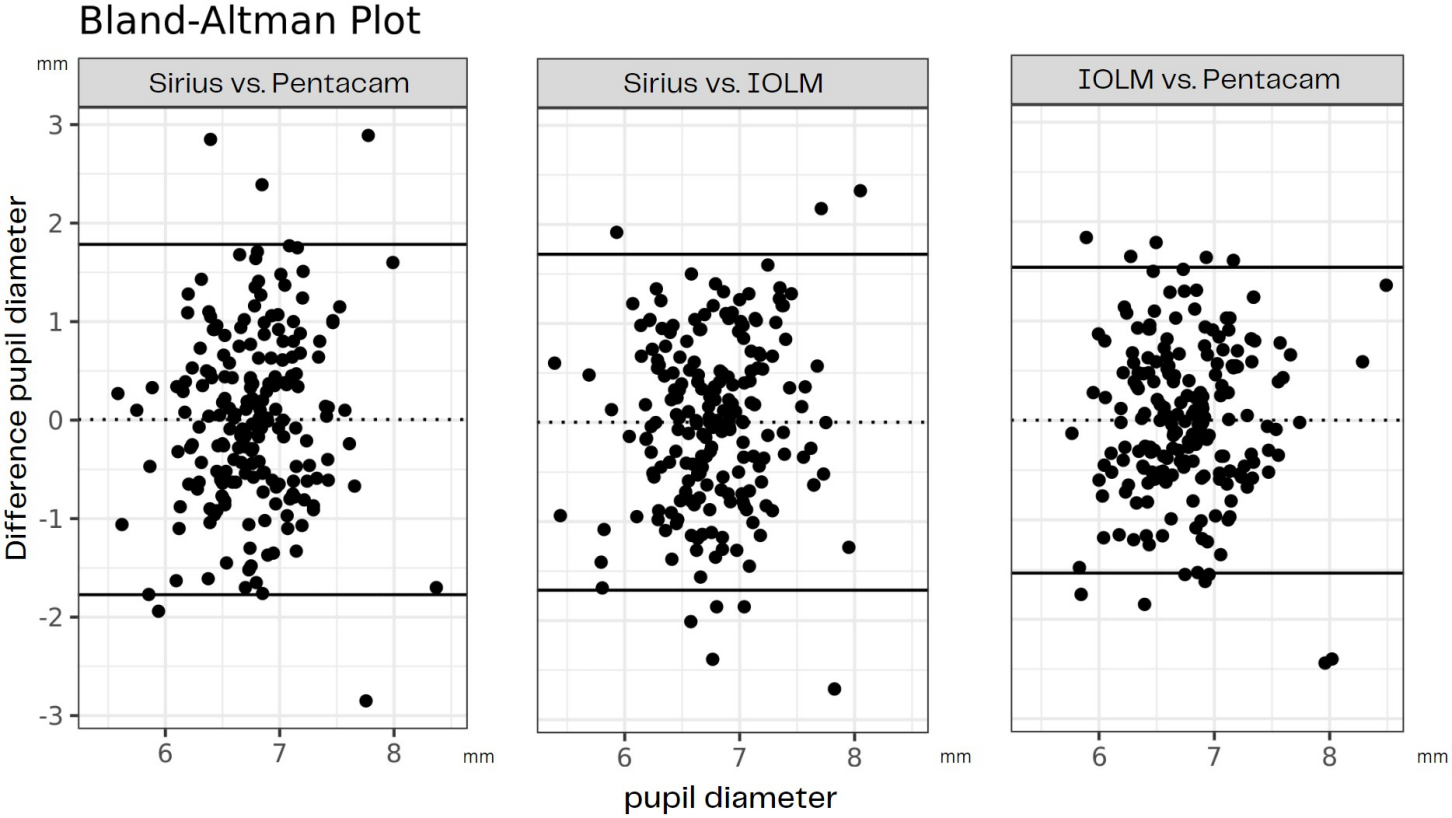
Bland-Altman for inter-device repeatability of device pairs.

Fig 2 shows the agreement for each pair of devices in a Bland-Altman plot. Table 5 lists the inter-device repeatability values for the device pairs. The agreement between the pairs of devices was low, with a very low ICC and high Sw. The mean PD value of the three devices was comparable (Table 1) and thus showed no significant difference between the scans (p > 0.05, Table 5).

**Table 5:** Inter-device repeatability of device pairs.

Statistic	Sirius vs Pentacam	Sirius vs IOLMaster	IOMaster vs Pentacam
**N Eyes**	204	204	204
**N Measurements**	408	408	408
**Mean difference**	0.01	0.00	0.00
**Difference between scans, p-value**	0.926	0.961	0.958
**Overall mean (mm)**	6.77	6.77	6.77
**Between SD (mm)**	0	0.13	0.22
**Within SD (mm)**	0.64	0.61	0.55
**ICC [95% CI]**	0 [0.00;0.06]	0.04 [0.00;0,17]	0.13 [0.00;0.26]
**LoA (mm)**	1.78,-1.77	1.7,-1.69	1.54,-1.54

**SD**: standard deviation; **ICC**: Intraclass Coefficient; **CI**: Confidence Interval; **LoA**: Limits of Agreement

## Discussion

The accurate determination of PD is one of the critical landmarks for patient selection prior to refractive surgery to achieve good refractive outcomes and reduce postoperative HOA. A mismatch between the effective optical zone and the scotopic pupil can lead to poor vision quality [6]. The precise measurement of PD is difficult because of its dynamic structure, also known as hippus [7]. Therefore, repeated measurements are crucial to minimize the inaccurate assessment of PD [8, 9].

Influencing factors include the different illuminations of both the room and of the fixation targets used for the different measuring devices. Likewise, the PD can vary depending on near-object accommodation and medication intake [10]. The patient’s age and emotional state are also influencing factors [11, 12]. Additionally, it seems that a binocular measurement of pupil size is closer to reality than a monocular measurement [13]. Certainly, the algorithms and software used in the different devices contribute to variations in PD readings [10].

A large scotopic PD has been reported in several studies as a risk factor that can lead to HOA and reduced contrast vision [14–16]. It was previously shown by others that depth-of focus decreases with larger pupil size [17–19]. After various keratorefractive procedures such as radial keratotomy (RK), photorefractive keratectomy (PRK) and LASIK, the occurrence of HOA and consequent decline in visual quality has been reported [20–22]. Although HOA are less common in SMILE patients, it is also important to create a large enough lenticule diameter in these eyes [23–25]. High PD also plays an important role in patients with phakic intraocular lenses, leading to significant HOA at night [26]. Therefore, the development of new phakic lenses aims to create a wider optical zone [27]. Likewise, depending on the IOL design, pseudophakic patients may suffer from dysphotopsia [28].

In addition, some studies demonstrated a change in PD after lens implantation. Some results suggest a decrease [29–31], while others propose a postoperative increase in PD, which makes the question of accurate PD measurement even more important [32].

However, PD is only one factor contributing to postoperative HOA. Additional factors like the Stiles-Crawford effect and other unknown factors that may also cause poor visual quality must be taken into account [33]. In this context, it must be said that flap-associated complications in LASIK patients, such as dry eye, are at least as important factors [34].

Since PD is an important inclusion or exclusion criterion in refractive surgery, the accuracy of its measurement is of high importance. However, only a few studies have addressed the measurement precision of PD so far. To the best of our knowledge, this is the first study presenting results on the repeatability of topographic measurements in young healthy patients.

The repeatability and reproducibility of infrared pupillometers, including hand-held instruments, have been confirmed in previous studies, although variations in pupil size at different time points have also been noted [35–37]. However, the values obtained by hand-held pupillometers seem to suffer under the subjective influence of the examiner, according to [8]. Therefore, topography systems for biometric assessment of the eye can provide more accurate results in planning keratorefractive interventions.

Previous studies have shown the high repeatability of PD determination with AL-Scan (Nidek Ltd., Gamagori, Japan) and good agreement with IOLMaster, even in children [38, 39]. In contrast, other groups found poor repeatability for PD measurements with the AL-Scan and the OA-2000 (Tomey, Nagoya, Japan) biometer [40, 41]. Altan et al. showed a high dispersion of PD values obtained with the Sirius topographer, especially under scotopic light conditions [10].

Although previous studies concluded that repeatability was high for most measured parameters (including PD) using the Pentacam system [42, 43], Shankar et al. showed poor repeatability in the measurement of PD, although under standardized test conditions [44]. Therefore, the authors do not recommend the use of its PD values for clinical purposes, e.g., to calculate the ablation zone. This is similar to the results we have obtained with the Pentacam HR. Another study showed the superiority of a slit-scanning corneal tomographer (Orbscan II, Bausch & Lomb, Rochester, NY) compared to the Pentacam Scheimpflug system in terms of accuracy of PD measurement [45]. Compared to another Scheimpflug-based tomography system (Galilei G4, Ziemer, Switzerland), the Pentacam showed higher PD values, which was considered clinically significant [46].

Our results show a moderate intra-device repeatability in the IOLMaster and Sirius system, but not with the Pentacam, which resulted in the largest variance (Sw) and, thus, the smallest precision (ICC). Comparing the three consecutive scans obtained with IOLMaster and Sirius, we observed a marginal reduction in pupil diameter from scan 1 to 3, indicating a potential adaptation to the subtle device illumination.

The results of the previous studies, together with our study, indicate poor agreement between the different devices (inter-device repeatability) and indicate that they are not interchangeable for the evaluation of PD.

Based on our results, the use of Sirius or IOLMaster or the combination of both devices seems reasonable in determining pupil size, in young patients. Alternatively, measurements with previously proven reliable pupillometers could help to determine the most appropriate optical zone [7]. Additionally, care should be taken to ensure uniform room lighting during the patient examinations. Adherence to a dark adaptation protocol can also help to minimize influencing factors [47].

The limitations of our study include the possibility of high correlation between right and left eye PD. Since our results are based on specific room conditions, varying clinical environments may result in different PD readings.

In summary, our results indicate higher repeatability in Sirius and IOLMaster compared to Pentacam when assessing the PD, which should be taken into account in the preoperative examination of refractive patients, especially in young adults and borderline cases.
